# C-Shaped Root Canal Morphology in Mandibular Posterior Teeth—A Retrospective CBCT Study Based on Two Classification Systems

**DOI:** 10.3390/diagnostics16193127

**Published:** 2026-09-26

**Authors:** Mubashir Baig Mirza, Abdulaziz Abdulwahed, Abdullah AlShehri, Ali Robaian AlQahtani, Mustufa Chawre, Osamah AlQasem, Shahad AlBader, Mohammed A. S. AbuelQomsan, Laila S. AlMufleh, Ahmed AlMokhatieb

**Affiliations:** 1 Conservative Dental Sciences, College of Dentistry, Prince Sattam bin Abdulaziz University, Al-Kharj 11942, Saudi Arabia; 2 School of Dental Sciences, Health Campus Universiti Sains Malaysia, Kubang Kerian 16150, Malaysia

**Keywords:** C-shaped canal, cone beam computed tomography, mandibular molars, premolar, Saudi subpopulation

## Abstract

**Background/Objectives:** Understanding the root canal morphology is crucial for successful endodontic therapy, particularly in teeth with anatomical variations such as C-shaped canals. These canals have an unusual shape, making clinical treatment challenging due to irregularities and inter-canal connections between roots. This study aimed to determine the prevalence of C-shaped root canals in mandibular posterior teeth in a Saudi subpopulation and to characterize their morphology using Fan’s classification and the Ahmed and Dummer classification system. The distribution of C-shaped canals was further described according to tooth type, age, gender, side, and bilateral occurrence. **Methods:** A retrospective cross-sectional study was conducted using 228 scans acquired from December 2023 to December 2025, comprising 1961 mandibular posterior teeth. Teeth were evaluated for C-shaped canals and classified using Fan’s classification at the coronal, middle, and apical thirds, as well as the Ahmed and Dummer classification system. Data were analyzed using descriptive statistics, the chi-square test, and Fisher’s exact test. Holm–Bonferroni correction was applied for exploratory comparisons, and Cramer’s V was used as an effect size measure. **Results:** The overall prevalence of C-shaped canals was 2.49% (49/1961 teeth). Mandibular second molars (MSMs) showed the highest percentage (8.2%), followed by third molars (3.6%), and first premolars (0.8%). First molars and second premolars did not exhibit such morphology. Age group was not clearly associated with C-shaped prevalence (*p* = 0.406; Cramer’s V = 0.059). Fan’s classification showed the predominance of C1 shape coronally and C3 type in the middle/apical thirds of second molars. The most common Ahmed and Dummer morphology was (CsC^III^)^2^ TN M/D (51%). No clear gender- or side-related pattern was observed. Tooth-level contralateral symmetry was observed in 24 out of 49 C-shaped teeth (49%). **Conclusions:** C-shaped root canal morphology was uncommon and occurred predominantly in MSMs. No gender-, side-, or age-related differences were observed. Since tooth-level anatomical data are presented, bilateral findings should prompt careful contralateral assessment and should not be interpreted as a conclusive indicator of bilateral occurrence.

## 1. Introduction

Effective endodontic treatment relies on understanding the internal anatomy and meticulously cleaning, shaping, and three-dimensionally (3D) filling the entire root canal space [1]. Posterior teeth are often more challenging to treat because of their complex anatomy, variations in canal types, and multiple root canals. This intricate anatomy can make it difficult to clean the entire root canal space, which may lead to endodontic treatment failure or persistent symptoms [2]. One such anatomical complication is the C-shaped roots formed by the incomplete fusion of Hertwig’s epithelial root sheath in the developing tooth, either buccally or lingually [3]. Its distinctive ribbon-shaped canal cross-section, abundant inter-canal communications, and deep radicular grooves make root canal therapy difficult and require special care to avoid complications such as strip perforation [4]. Due to these complexities, clinicians must rely on instruments and techniques that ensure controlled contact with the canal walls during instrumentation, adequate disinfection, and the use of bioceramic or thermoplasticized gutta-percha for a 3D seal [5].

Root canal anatomy evaluation has progressed from traditional approaches such as periapical radiographs and clearing/staining methods [6]. Currently, micro-CT and cone beam computed tomography (CBCT) are essential tools for analyzing root canal structures in three dimensions [7]. While the former remains the gold standard technique, its invasiveness makes it impractical in clinical practice [8]. In contrast, CBCT gained recognition because it is non-invasive and allows the visualization of the anatomy in the axial, sagittal, and coronal views. Moreover, because it uses relatively low radiation doses, in accordance with the “As Low As Reasonably Achievable” principle and small field-of-view (FOV) protocols, it can be used for endodontic diagnostics [9].

The prevalence of C-shaped canal systems varies considerably across populations. Epidemiological studies have reported the highest incidence rates in East Asian populations (up to 36–43.1%) [10]. Mandibular second molars (MSMs) are the most common teeth, followed by maxillary first molars. Other posterior teeth have also been reported to exhibit C-shaped root canal morphology to a lesser extent [11]. Previous studies have also reported bilateral C-shaped canals, particularly in MSMs, with rates ranging from 63% to 85.9% [12,13].

Because traditional root canal morphology classifications, including Vertucci’s classification, cannot describe C-shaped root canals, Melton et al. developed a classification dividing them into type I (continuous), type II (semicolon-shaped), and type III (two or three separate canals) [14]. Fan et al. introduced additional types of C-shaped canal morphology, including type IV (round/oval canals) and type V (canals with unidentifiable lumens, especially apically) [3]. Although these classifications can help describe the canal configuration at different anatomical levels (coronal, middle, and apical), they are insufficient to describe factors such as inter-canal communications, which root is affected, and the possibility of root fusion. Therefore, Ahmed and Dummer suggested a CBCT-based coding system that includes information on the number of teeth, roots, and canal configurations [15]. This system includes the abbreviation “Csc” for C-shaped canals, a superscripted roman numeral indicating the C-type morphology, and a superscripted number after the bracket indicating the number of roots. It further includes a two-digit FDI tooth number and the initial of the root (e.g., (M) mesial and (D) distal for a two-rooted tooth), with in-between signs such as (“/”) for root fusion and (“//”) for inter-root communication [15].

Although studies from Saudi Arabia have reported the prevalence of the C-shaped canal, further research remains necessary, given regional variations observed within the same populations in Iran and India [16,17,18]. This is the first study to evaluate the C-shaped morphology in this cohort from the central region of the Kingdom. Additionally, it is the first to evaluate its presence across all mandibular posterior teeth using the anomaly-based classification. Thus, this study aims to (1) assess the prevalence of C-shaped root canals in mandibular premolars and molars using CBCT in the Saudi subpopulation; (2) classify C-shaped canals using the Fan classification scheme at various anatomical levels combined with Ahmed and Dummer coding; (3) describe tooth-level bilateral occurrence; and (4) evaluate their distribution according to gender and age.

## 2. Materials and Methods

### 2.1. Study Design and Setting

This cross-sectional observational study analyzed CBCT scans acquired between December 2023 and December 2025 to assess C-shaped root canals in mandibular posterior teeth. The protocol received approval from the Standing Committee of Bioethics Research (SCBR) (Ref. No. SCBR-161/2023) and complied with the principles of the Declaration of Helsinki. The manuscript follows the STROBE reporting guidelines and includes a completed STROBE checklist, provided as Supplementary Table S1. In accordance with the institutional protocol, informed consent was obtained and documented for all patients receiving treatment at the College of Dentistry, Prince Sattam bin Abdulaziz University (PSAU). This consent permits the use of their data, including radiographs, for educational and research purposes. To protect participant confidentiality, all data were anonymized.

### 2.2. Sample Size and Data Collection

The sample size for this retrospective study was based on the availability of CBCT scans in the PSAU institutional database during the study period. A total of 430 CBCT scans performed from December 2023 through December 2025 were screened. Based on the inclusion/exclusion criteria, a sample of 228 scans (142 female, 86 male) was selected for evaluation. The sample consisted of 1961 teeth, including 388 mandibular first premolars (MFPs), 403 mandibular second premolars (MSPs), 423 mandibular first molars (MFMs), 416 MSMs, and 331 mandibular third molars (MTMs). The discrepancy in the number of teeth analyzed is due to incomplete sets of teeth in the CBCT records. An expected prevalence of 10% was selected based on previously reported prevalence estimates among MSMs in comparable regional populations, including Yemen, Sudan, Jordan, Lebanon, and Pakistan. Using an expected prevalence of 10%, a 95% confidence level, and a 5% margin of error, the minimum required sample size was calculated to be 139 patients. The final sample consisted of 228 patients/scans, exceeding this estimate. However, because multiple mandibular posterior teeth were assessed in the same patient, tooth-level analyses should be interpreted as descriptive anatomical variations rather than as fully independent patient-level data.

### 2.3. Inclusion and Exclusion Criteria

CBCT scans were included if they met the inclusion criteria, which included an age range of 18 to 65 years, the presence of teeth that had not undergone endodontic treatment, and the absence of crowns or large metallic restorations. CBCT scans were excluded when image quality compromised reliable identification of root canals due to motion-related artifacts or interference affecting the tooth. Additionally, scans were excluded if voxel or slice reconstruction hindered assessment in all three dimensions, or if insufficient contrast or resolution prevented delineation of the canal outlines. Teeth with root deformities, open apices, and root resorption were also excluded from the study.

### 2.4. CBCT Procedures and Measurements

The CBCT scans at the dental college are conducted using the Carestream CS9300 (Carestream Dent LLC., Atlanta, GA, USA), featuring a voxel size of 0.09 mm. The system operates at 60–90 kV and 2–15 mA, with a scanning time of 10 s and a field of view (FOV) of 10 × 10 cm. Analysis was performed with the CS 3D Imaging software V8.0.24 (Carestream Dental LLC., Atlanta, GA, USA) in the axial, coronal, and sagittal multiplanar reconstruction views. Axial sections were evaluated sequentially from the canal orifice to the apex. Fan’s classification was recorded at three standardized root levels: 2 mm below the canal orifice for the coronal third, at the midpoint of the root for the middle third, and 2 mm above the apex for the apical third. Ahmed and Dummer coding was applied according to tooth number, number of roots, root fusion, canal configuration, and inter-root or inter-canal communication.

### 2.5. Observer Calibration

To minimize observer bias, calibration was conducted over three weeks before the study. This calibration and reliability check used 50 CBCT scans, all of which were part of the final sample. The observers remained blinded during the evaluation. Experienced endodontists M.B.M. and A.A., each with 8 years of CBCT interpretation experience, and A.A.M., with 6 years of CBCT interpretation experience, trained and calibrated the reviewers (O.A., S.A., and L.A.) to ensure consistent identification and classification of canal morphologies. For intra-observer reliability, the same 50 scans were re-evaluated after a 2-week interval under the same viewing conditions. Inter- and intra-examiner agreement was assessed using Cohen’s kappa coefficient. Excellent reliability was observed, with an inter-examiner kappa of 0.91 and an intra-examiner kappa of 0.87, indicating an almost perfect agreement. Disagreements during the study were resolved through consensus among the observers and supervising endodontists.

### 2.6. Statistical Analysis

Data were analyzed using SPSS software version 21.0 (SPSS Inc., Chicago, IL, USA). Descriptive statistics were used to report frequencies and percentages. The distribution of C-shaped canal morphology was summarized according to tooth type, age group, gender, side, Fan’s classification, and Ahmed and Dummer coding. Age was categorized into five groups: 18–24, 25–34, 35–44, 45–54, and 55–64 years. Fan’s classification at the coronal, middle, and apical thirds was reported descriptively because these levels represent repeated observations within the same tooth.

When exploratory comparisons were retained, the association between categorical variables was assessed using Pearson’s chi-square and Fisher’s exact tests when assessing small cell counts. Holm–Bonferroni correction was applied to the resulting *p*-values for multiple exploratory comparisons. Cramer’s V was used as the effect size measure. Because tooth-level observations were clustered and the number of identified C-shaped cases was limited, multivariable regression modeling was not performed. A *p*-value of less than 0.05 was considered statistically significant for exploratory analyses.

## 3. Results

### 3.1. Prevalence

A total of 1961 mandibular posterior teeth were analyzed. Overall, 49 teeth showed C-shaped root canal morphology, giving a descriptive tooth-level prevalence of 2.49%, with the highest frequency in MSMs (8.2%), followed by MTMs (3.6%) and MFPs (0.8%). No C-shaped canal morphology was identified in MFMs and MSPs. Table 1 summarizes the descriptive prevalence of C-shaped canals and their exploratory distribution according to gender and side.

### 3.2. Fan’s Classification

For Fan’s classification, assigning a classification to the tooth per se is not feasible, as the canal configuration may vary along its length. Therefore, the configuration is described at three standardized root levels for each tooth, in line with the study conducted by AlOmari et al. among the Jordanian population (2 mm below the orifice for the coronal third, at the middle of the root for the middle third, and 2 mm above the apex for the apical third, measured on the CBCT) [19]. Figure 1 illustrates the distribution of Fan classification types (C1–C4) across the coronal, middle, and apical root levels of the mandibular posterior teeth exhibiting C-shaped canal morphology.

Stacked bars depict the percentage distribution of Fan classification types (C1–C4) within each tooth group. These observations represent tooth-level assessments and are summarized descriptively rather than being analyzed as independent categories. In the MSMs, C1 is predominant at the coronal levels (73.5%), whereas C3 is more common in the middle and apical thirds (41.2% and 47.1%, respectively). C4 configuration also increases markedly in the apical third (17.6%). MTM shows a relatively balanced C-shaped configuration, with a higher prevalence of C1 configuration across the thirds (50–58.3%). C2-type configuration was more commonly seen in the middle and apical thirds of the MFPs (66.7%). Figure 2 shows axial CBCT images depicting Fan’s classifications at different levels.

### 3.3. Ahmed and Dummer Classification

C-shaped teeth were classified using the Ahmed and Dummer classification. The majority of teeth were classified as (CsCIII)^2^TN M/D group (*n* = 25), indicating that most mandibular posterior teeth with C-shaped anatomy were two-rooted, with fused roots encasing a type III (C3) canal morphology featuring separate, asymmetrical mesial and distal canals, with the distal canal seemingly wider than the mesial. Additionally, the (CsCI)^2^TN M//D group of C-shaped canal morphology was also observed in higher numbers, particularly in the third molars, indicating two intercommunicating roots with a type I (C1) canal that merges into one before exiting the apex. Figure 3 shows CBCT views of C-shaped teeth depicting the Ahmed and Dummer classification.

The descriptive distribution of Ahmed and Dummer morphologies by tooth type are shown in Table 2.

Table 3 summarizes exploratory tooth-level comparisons of Ahmed and Dummer classification patterns by side and gender. Because multiple teeth were evaluated from the same patient, these comparisons are presented descriptively. No definitive side- or gender-related pattern was inferred from these exploratory comparisons.

Table 4 summarizes the age-wise distribution for the tooth groups in which C-shaped canal morphology was identified. C-shaped canals were observed across all age groups, with the highest number of cases seen in the 45–54 age group. The exploratory tooth-level analysis showed no statistically significant association between age group and C-shaped canal prevalence, χ^2^ = 3.999, df = 4, *p* = 0.406, with a very small effect size, Cramer’s V = 0.059.

Tooth-level contralateral symmetry of C-shaped teeth was observed in 12 cases, representing 24 of the 49 C-shaped teeth (49%). Of these, three scans involving MSMs showed identical C-shaped morphologies based on Ahmed and Dummer’s classification, accounting for six teeth, whereas the remaining nine scans, accounting for 18 teeth, showed non-identical C-shaped morphologies on the contralateral side, as shown in Table 5. Table 5 summarizes tooth-level contralateral symmetry among identified C-shaped teeth and does not represent true bilateral prevalence among all patients with both contralateral teeth present and evaluable.

Among patients with C-shaped MSMs, nine had bilateral cases (18 teeth). For MTMs, two patients had bilateral involvement (four teeth). One MFP patient also had bilateral involvement (two teeth). In total, 12 bilateral tooth pairs (24 teeth) were observed, as shown in Table 6.

## 4. Discussion

The present study evaluated the prevalence, morphological classification according to two systems, bilateral occurrence, gender, age, and side-wise distribution of C-shaped root canals in mandibular premolars and molars using CBCT. The overall prevalence of C-shaped canal configurations in mandibular posterior teeth is reported at 2.49% (49 out of 1961 teeth). Specifically, MSMs exhibit the highest prevalence at 8.2%, while MTMs and MFPs show prevalence rates of 3.6% and 0.8%, respectively. According to Fan’s classification, C1 morphology was most prevalent in the coronal region for all mandibular posterior teeth. C3 was more commonly observed in the middle and apical thirds of MSMs, C1 in the middle and apical thirds of MTMs, and C2 in the middle and apical thirds of MFPs. These observations were summarized descriptively because the coronal, middle, and apical measurements represent repeated assessments within the same tooth rather than independent observations. According to Ahmed et al., (CsC^III^)^2^TN M/D, indicating type III C-shaped canal morphology in a fused two-rooted tooth, was the most common. It was followed by (CsC^I^)^2^TN M//D, representing type I C-shaped canal morphology in a two-rooted tooth with intercommunications. Exploratory side- and gender-based distributions were not interpreted as definitive inferential findings because patient-level clustering was not modeled. Tooth-level contralateral symmetry was observed in 12 tooth pair cases, for 24 of the 49 C-shaped teeth.

C-shaped root canals have been documented in posterior maxillary and mandibular teeth, with a notably higher prevalence in MSMs [11,20]. In our study, we identified an 8.2% prevalence of C-shaped canals among MSMs, which closely aligns with the global prevalence of approximately 12–13.8% [10]. Notably, East Asian countries exhibit significantly higher prevalence rates, often regarded as normal rather than anomalous, ranging from 36% to 43.1%, with some reports indicating figures as high as 48.7% [10,21]. Reasonably high prevalence has also been reported in Central and South American countries like Ecuador 28%, Mexico 14.1%, and Brazil 14.32% [12,22,23]. Our findings are consistent with the reported prevalence rates in other Arab nations, including Yemen 9% [20], Sudan 10% [24], Jordan 10–12% [19,25], and Lebanon 9.09% [26], and lower than those observed in the Egyptian 12.8% [27], Kuwaiti 13% [28], and Emirati 17.9% [8] populations. In the West Asian region, our results are lower than those reported in Iraq 17.4% [29], Iran 17.6–21.4% [30,31], and Pakistan 10% [32]. Compared with MSMs, the MFM shows a lower incidence of C-shaped morphology. Reported incidence rates include 0.55% in the Chinese population [11], 2.39% in Brazil [23], 1.01% in Lebanon [26], 0.85% in the Turkish population [33], and 1.7% in Iran [34]. In our investigation, none of the 423 teeth examined exhibited C-shaped morphology, consistent with previous studies in Jazan and Yemen [16,35].

C-shaped morphology in an MTM is less frequently documented, primarily because few studies have examined its root canal morphology [36,37]. A plausible reason is that most pulpally involved third molars are extracted because of treatment challenges or because they are not considered strategically important in the treatment plan [36]. However, for restorative reasons, preserving these teeth through root canal treatment underscores the need for a comprehensive understanding of their morphology and associated anomalies [37]. In this investigation, we observed a prevalence of 3.6%, marginally higher than the 1.87% reported in a previous study from Jordan [36]. However, a study from India reported C-shaped root canals in approximately 12% of the population [37]. This study is, to our knowledge, the first to detail the prevalence of C-shaped morphology in MTMs in Saudi Arabia. Similarly, other multi-rooted teeth, such as premolars, have also been reported to have C-shaped anomalies [11]. In the present study, only three teeth showed C-shaped morphology in the MFP, and none were found in the MSP. Previously, Mashyakhy et al. in the Jazan region of Saudi Arabia reported a prevalence of C-shaped canals of 1.5% for the MFP and 0.8% for the MSP [16]. These differences may reflect genetic and ethnic variation within the same population, as well as methodological factors such as CBCT voxel size and image interpretation protocols, which can also influence prevalence estimates across studies. Differences within the same populations also underscore the need for more region-specific, large-scale studies to better understand intrapopulation variability. Reports indicate that 1.1% of the Chinese population has C-shaped premolars, with a higher figure of 16.9% reported in a micro-CT study [38]. The global prevalence of C-shaped MFPs has been reported to range from 0 to 28.94% [39,40]. Our results align with this range of globally reported data. These observations for the mandibular teeth evaluated in this study underscore the substantial impact of genetic and ethnic backgrounds on root canal morphology.

Fan’s classification offers a detailed analysis of the C-shaped anomaly, highlighting variations by length along the entire root canal [20]. Most of the existing literature has used this classification and its modifications to identify and classify C-shaped morphologies. In our study, in MSMs, C1 type was most common in the coronal third, followed by C3. In the middle third, C3 was more prevalent, followed by C1; in the apical third, C3 and C2 were more frequent, with an increased presence of C4. Our results closely match those observed in the Emirati population, where C1 was more prominent in the coronal third, and C3 was more common in the middle and apical thirds [8]. Conversely, in previous studies from Saudi Arabia and Yemen, C3 was the most common across various canal lengths [16,20]. Regarding MTMs, although two studies report the presence of C-shaped canals, additional morphological details, such as classification type, were not apparent in either study [36,37]. In mandibular premolars, the C2-type Fan’s classification was the most common at all levels (40.7%), with the C4 type observed exclusively in the coronal thirds within the Saudi subpopulation in Jazan [16]. In our study, type C2 was more prevalent in the middle and apical thirds, whereas type C1 predominated in the coronal third; type C4 was identified exclusively in the apical third. Nonetheless, given the low prevalence of C-shaped premolars in our study, such comparisons should be interpreted cautiously. Although the Fan’s classification holds up well, classifying at different depths becomes cumbersome, especially when inter-group or inter-study comparisons are needed [15]. In contrast, Ahmed and Dummer’s anomaly classification relies on the assessment of CBCT scans to provide a more comprehensive, tooth-specific approach, incorporating details on root number and fusion, as well as C-shaped root canal morphology, into a single code, thereby enhancing research efforts by facilitating comparative analysis [15,41]. This study is the first attempt to utilize this classification to represent C-shaped root canal morphology. All teeth identified as C-shaped had two roots, with 51% classified as (CsC^III^)^2^TN M/D.

Gender can also affect the prevalence of certain anatomical features [10], and differences in root canal morphology based on gender have been documented in the literature [42]. In the current study, although more C-shaped teeth were observed in female patients, the study sample itself included more female scans than male scans. Descriptively, no clear gender-based pattern emerged. This is consistent with previous reports from Jordan, Yemen, and Egypt [19,20,27]. Similarly, in previous Saudi subpopulations, Mashyakhy et al. and Al-Fouzan et al. reported no significant gender differences in the prevalence of C-shaped canals [16,18]. In contrast, some studies from West Asian and South American populations have reported higher prevalence among females [8,12,22,28,29,32], whereas a study from Eastern China reported male predilection in premolars [11]. Overall, the available findings suggest that population and tooth type may have a stronger influence on C-shaped canal morphology than gender alone.

The age-wise analysis showed that C-shaped canals were present across all age groups, with the highest cases in the 45–54-year group and fewer cases in the 18–24- and 55–64-year groups. These findings are consistent with a study by Martins et al. in the Portuguese population, in which the highest prevalence was also in the 45–54 age group and the lowest among patients over 65 [43]. However, because C-shaped canal morphology is primarily developmental, age is unlikely to determine its presence. In this exploratory analysis, we found no statistically significant association between age and the occurrence of C-shaped canals, consistent with findings in the Korean population [44]. Conversely, another Korean study reported significant differences between individuals aged 61 and older and those aged 21–30 [13]. Nonetheless, age-related changes such as secondary dentin deposition, canal narrowing, and pulp canal obliteration may still affect canal morphology.

Existing research has well-established bilateral C-shaped canal morphology in MSMs, with multiple studies indicating a tendency for C-shaped canals to occur on both sides of the mandible [10,11]. In this study, 24 of 49 C-shaped teeth (49%) showed contralateral symmetry at the tooth level, slightly higher than the 40.7% reported in Jordan [19] but lower than the 60% in Lebanon [26] and 63% in Ecuador [12]. Studies by Zheng et al. and Kim et al. reported even higher bilateral symmetry in MSMs among Chinese and Koreans, at 82% and 81%, respectively [45,46]. Clinically, a C-shaped canal on one side warrants careful assessment of the contralateral tooth. Furthermore, the complex anatomy of C-shaped canal systems, including fins, isthmuses, and inter-canal communications, may complicate cleaning, shaping, and obturation procedures. In such cases, CBCT imaging can assist with diagnosis and treatment planning, while enhanced irrigation protocols, careful instrumentation given thinner dentinal walls, and obturation techniques using bioceramic sealers or thermoplasticized gutta-percha may improve adaptation to irregular canal spaces and facilitate three-dimensional filling of the root canal system [5,12].

This study has several limitations. The retrospective, single-center design may introduce selection bias and limit generalizability. Multiple teeth from the same patient were evaluated, introducing potential within-patient correlation. Although the sample size calculation was appropriately based on patient-level observations, tooth-level analyses were not adjusted for intraclass correlation. Consequently, the reported tooth-level prevalence estimates and confidence intervals should be interpreted as approximate descriptive measures rather than fully independent estimates. Therefore, tooth-level findings should be viewed primarily as anatomical observations rather than patient-level estimates. Given the descriptive aims of the study and the limited number of C-shaped cases, formal clustered statistical modeling, such as generalized estimating equations (GEEs) or mixed-effects logistic regression, was not performed. Consequently, potential associations between demographic variables and C-shaped canal morphology should be interpreted cautiously. Fan’s classification was recorded at the coronal, middle, and apical thirds of the same tooth; therefore, these observations represent repeated measures and were summarized descriptively. As formal sensitivity analysis was not performed, missing teeth were not analyzed as independent predictors, which may have affected tooth-level prevalence, particularly in MTMs. Side and bilateral analyses were descriptive and were not based on formal paired models or denominators restricted to all patients with both contralateral teeth present and evaluable. Additionally, the study was limited to CBCT-based assessment, which, while highly accurate, cannot provide the histological resolution of micro-CT studies. The relatively small number of identified C-shaped cases limited the statistical power of subgroup analyses. Finally, the study was confined to a single geographic region of Saudi Arabia, and the results may not be generalizable to the entire Kingdom. Future multicenter prospective studies incorporating micro-CT analysis and histological validation are recommended to support these findings. Future multicenter studies should consider incorporating design effect adjustment, standardized clinical variables, and use advanced multivariable and clustered analysis techniques, such as GEEs or mixed-effects models, to better assess how confounding factors and within-patient correlations influence outcomes.

## 5. Conclusions

Within the limitations of this CBCT study, C-shaped root canal morphology was relatively uncommon (2.49%) in mandibular posterior teeth in this Saudi subpopulation, with the highest prevalence observed in MSMs (8.2%). Fan’s classification showed a predominance of C1 coronally and C3 in the middle and apical thirds, while Ahmed and Dummer’s classification identified (CsC^III^)^2^TN M/D as the most common pattern. No clear age-, gender-, or side-related pattern was observed. Tooth-level contralateral symmetry was observed in 49% of identified C-shaped teeth; however, this descriptive finding should be interpreted cautiously and should not be interpreted as definitive evidence of bilateral occurrence.

## Figures and Tables

**Figure 1:**
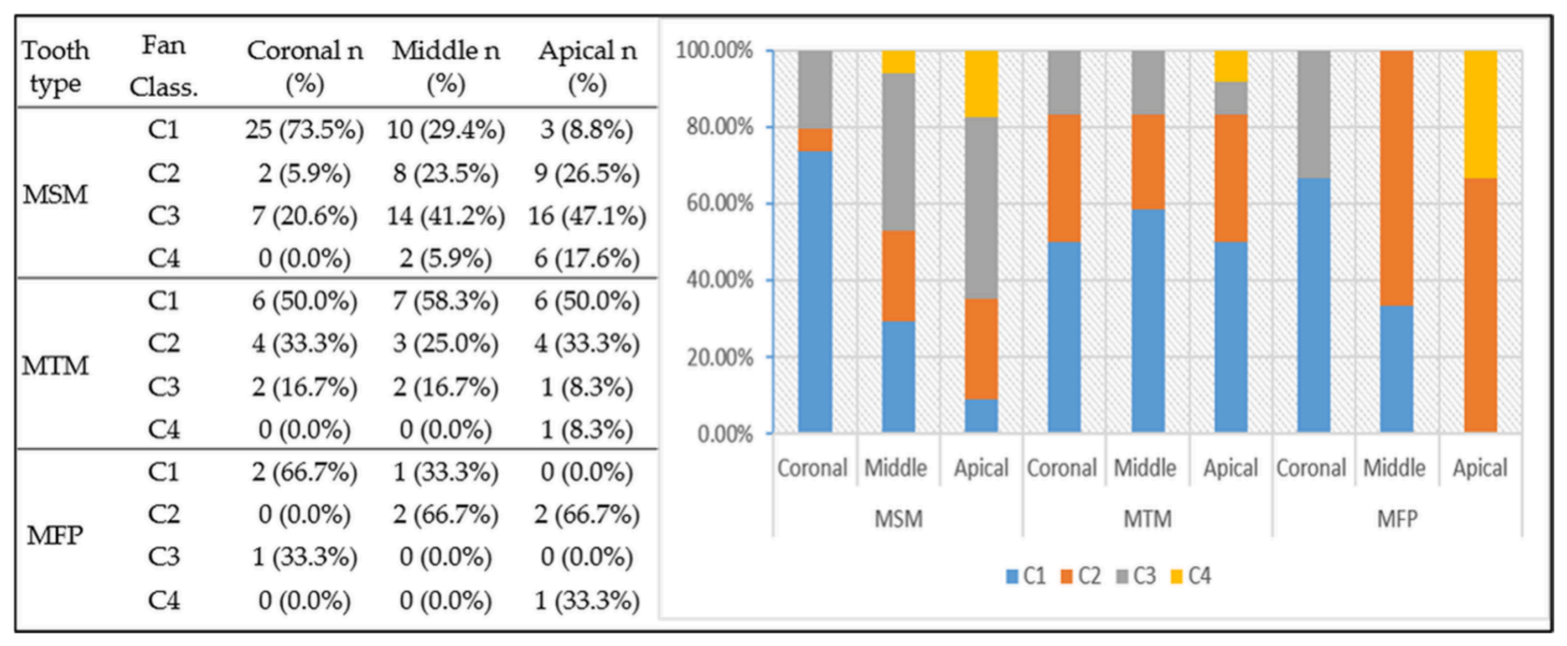
Fan’s classification across root levels in C-shaped mandibular posterior teeth. MFP, mandibular first premolar; MSM, mandibular second molar; MTM, mandibular third molar; Fan’s classification C1–C4 for coronal, middle, and apical thirds.

**Figure 2:**
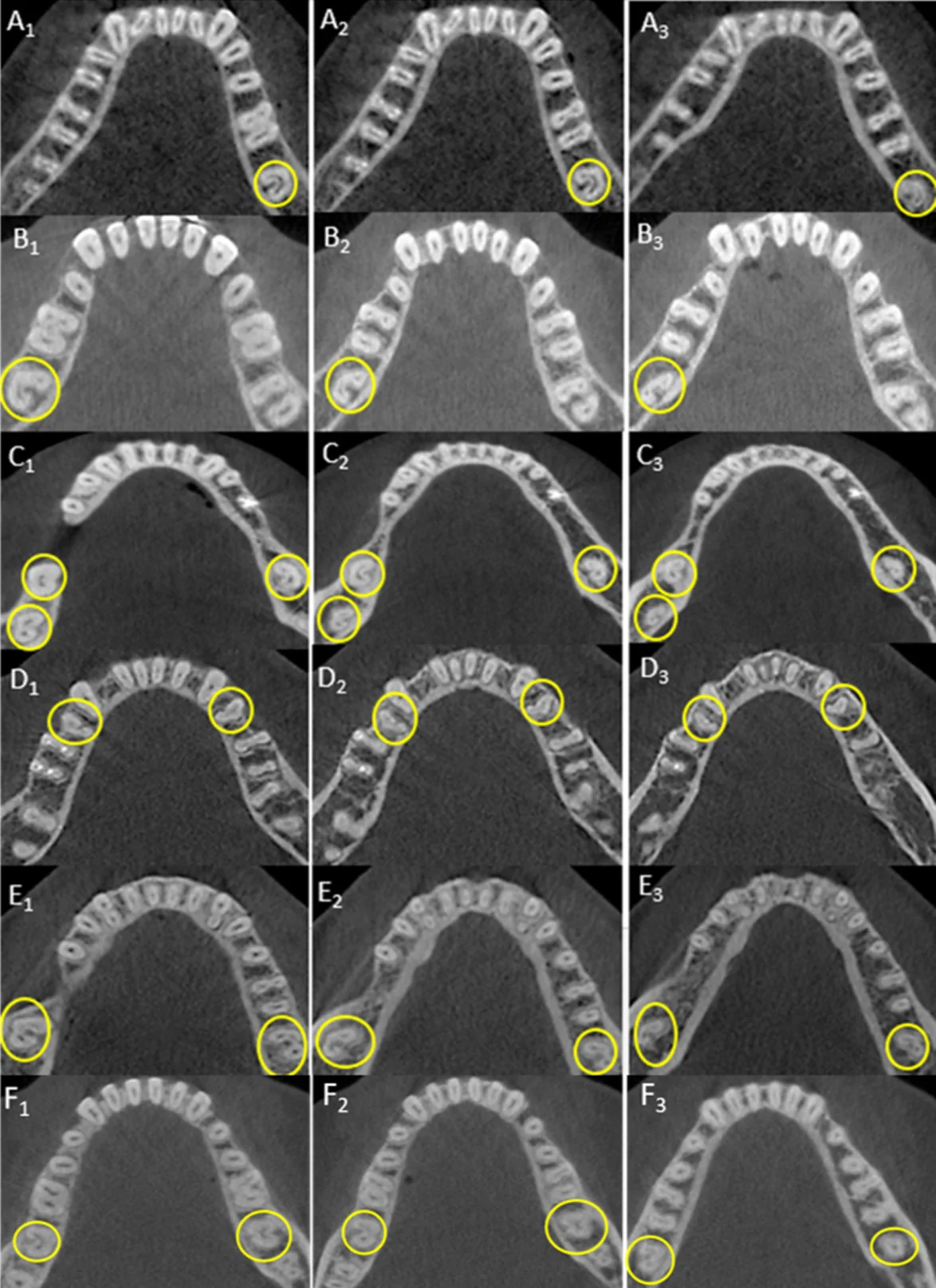
Representative axial CBCT images showing Fan’s classification of C-shaped canal morphologies at different root levels. Views of the mandibular jaw in axial CBCT: 1, coronal third; 2, middle third; and 3, apical third. (**A_1_**–**A_3_**) show the left mandibular second molar (MSM) with C1 canal morphology in the coronal third, and C2 canal morphology in the middle and apical thirds. (**B_1_**–**B_3_**) show the right MSM with C2 canal morphology in the coronal third and C3 morphology in the middle and apical thirds; (**C_1_**–**C_3_**) show bilaterally symmetrical C-shaped MSMs with C1 morphology in the coronal and middle thirds, and C2 morphology in the apical third on the right side, while the left MSM shows C2 morphology in the coronal third, C3 morphology in the middle and apical thirds; (**C_1_**–**C_3_**) also show mandibular third molars (MTMs) with C3 morphology in the coronal and middle thirds and C2 in the apical third; (**D_1_**–**D_3_**) show mandibular first premolars (MFPs) with bilaterally symmetrical C-shaped teeth with C3 morphology in the coronal third, C2 morphology in the middle and apical thirds on the right side, while the left MFP shows C1 morphology in the coronal and middle thirds and C2 morphology in the apical third. (**E_1_**–**E_3_**) show bilaterally symmetrical MSMs with C2 morphology in the coronal third and C3 in the middle and apical thirds (identical). (**F_1_**–**F_3_**) show bilaterally symmetrical C-shaped MSMs with C1 morphology in the coronal third, C2 morphology in the middle third, and C3 in the apical third on the right MSM, while the left MSM shows C1 morphology in the coronal and middle thirds and C4 morphology in the apical third.

**Figure 3:**
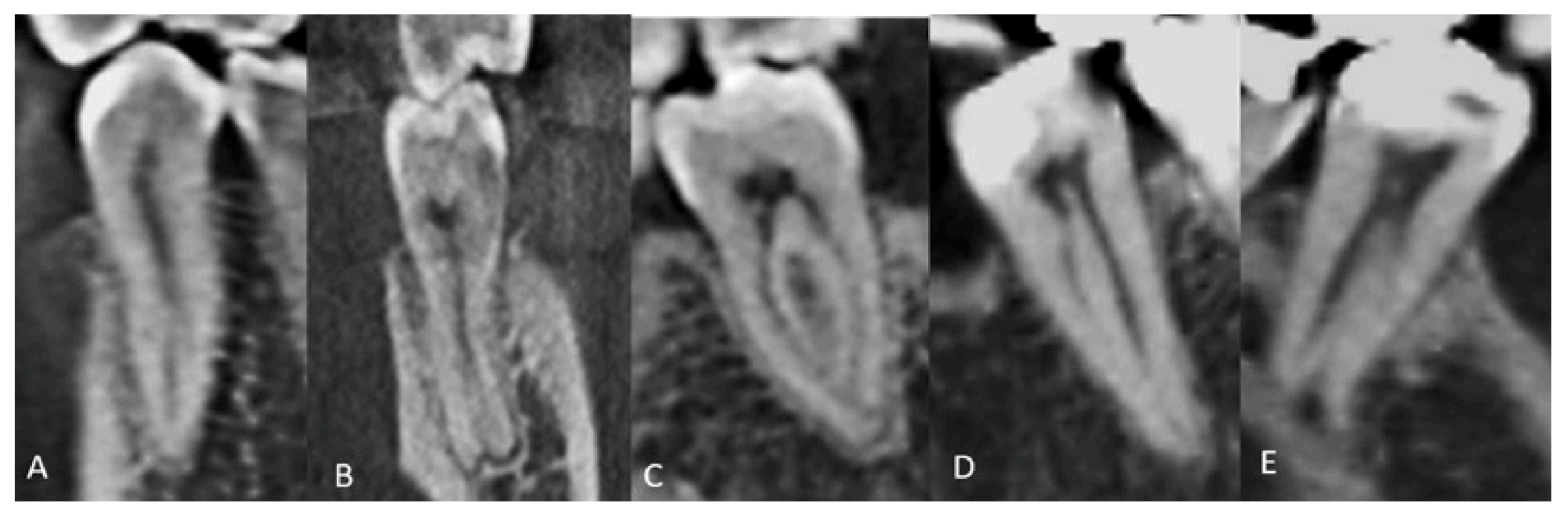
Ahmed and Dummer classification. (**A**): (CsC^I^)^2^TN M//D, C-shaped premolar with type I C-shaped morphology with fused roots and intercommunication; (**B**): (CsC^III^)^2^TN M/D, C-shaped premolar with type III C-shaped morphology with fused roots; (**C**): (CsC^I^)^2^TN M//D, C-shaped molar with type I C-shaped morphology with fused roots and intercommunication; (**D**): (CsC^II^)^2^TN M/D, C-shaped molar with type II C-shaped morphology with fused roots; (**E**): (CsC^III^)^2^TN M/D, C-shaped molar with type III C-shaped morphology with fused roots.

**Table 1:** Descriptive tooth-level prevalence and exploratory distribution of C-shaped canals according to tooth type, gender, and side.

Tooth Type	Total Teeth (*n*)	C-Shaped Teeth n (%)	95% CI	Male *n*/*N* (%)	Female *n*/*N* (%)	*p*-Value (Adj *p*)	Cramer’s V	Right *n/N* (%)	Left *n/N* (%)	*p*-Value (adj *p*)	Cramer’s V
MFP	388	3 (0.8)	0.26–2.25	0/152 (0.0)	3/236 (1.27)	0.283 (1.000)	0.071	1/184 (0.54)	2/204 (0.98)	1.000 (1.000)	0.025
MSM	416	34 (8.2)	5.91–11.20	12/151 (7.95)	22/265 (8.30)	1.000 (1.000)	0.006	19/214 (8.90)	15/202 (7.40)	0.721 (1.000)	0.027
MTM	331	12 (3.6)	2.09–6.23	4/173 (2.31)	8/158 (5.06)	0.242 (1.000)	0.074	5/155 (3.23)	7/176 (3.98)	0.776 (1.000)	0.020

MFP, mandibular first premolar; MSM, mandibular second molar; MTM, mandibular third molar; n, total number of C-shaped teeth; N, total number of teeth; CI, confidence interval. *p*-value for Fisher’s exact test with significance at <0.05; adj *p*, adjusted *p*-value after Holm–Bonferroni adjustment. Cramer’s V was used as the effect size measure.

**Table 2:** Distribution of the C-shaped canal configuration according to Ahmed and Dummer classification.

Ahmed’s Classification	*n* (%)	MFP *n* (%)	MSM *n* (%)	MTM *n* (%)
(CsC^I^)^2^TN M//D	16 (32.7)	1 (2)	9 (18.4)	6 (12.3)
(CsC^II^)^2^TN M/D	8 (16.3)	0	7 (14.3)	1 (2)
(CsC^III^)^2^TN M/D	25 (51)	2 (4.1)	18 (36.7)	5 (10.2)
Total	49 (100)	3 (6.1)	34 (69.4)	12 (24.5)

(CsC^I^)^2^TN M//D, C-shaped teeth with type I C-shaped morphology with fused roots and intercommunication; D: (CsC^II^)^2^TN M/D, C-shaped teeth with type II C-shaped morphology with fused roots; (CsC^III^)^2^TN M/D, C-shaped teeth with type III C-shaped morphology with fused roots; MFP, mandibular first premolar; MSM, mandibular second molar; MTM, mandibular third molar; n, number of teeth. Note: Percentages are calculated from the total number of identified C-shaped teeth (n = 49). Values are descriptive tooth-level findings.

**Table 3:** Descriptive distribution of C-shaped canal morphologies based on Ahmed and Dummer classification patterns by side and gender.

Tooth Type	Ahmed’s Classification	Right *n* (%)	Left *n* (%)	Male *n* (%)	Female *n* (%)
MFP	(CsC^III^)^2^TN M/D	1 (2)	1 (2)	0	2 (4.1)
	(CsC^I^)^2^TN M//D	0	1 (2)	0	1 (2)
MSM	(CsC^III^)^2^TN M/D	11 (22.4)	7 (14.3)	6 (12.2)	12 (24.5)
	(CsC^II^)^2^TN M/D	6 (12.2)	1 (2)	2 (4.1)	5 (10.2)
	(CsC^I^)^2^TN M//D	2 (4.1)	7 (14.3)	4 (8.2)	5 (10.2)
MTM	(CsC^III^)^2^TN M/D	2 (4.1)	3 (6.1)	2 (4.1)	3 (6.1)
	(CsC^II^)^2^TN M/D	0	1 (2)	0	1 (2)
	(CsC^I^)^2^TN M//D	3 (6.1)	3 (6.1)	2 (4.1)	4 (8.2)

(CsC^I^)^2^TN M//D, C-shaped teeth with type I C-shaped morphology with fused roots and intercommunication; (CsC^II^)^2^TN M/D, C-shaped teeth with type II C-shaped morphology with fused roots; (CsC^III^)^2^TN M/D, C-shaped teeth with type III C-shaped morphology with fused roots; MFP, mandibular first premolar; MSM, mandibular second molar; MTM, mandibular third molar; n, number of teeth; TN, tooth number according to the FDI two-digit tooth numbering system. Percentages are calculated from the total number of identified C-shaped teeth (*n* = 49). Values are descriptive tooth-level findings and were not adjusted for patient-level clustering.

**Table 4:** Age-wise distribution of C-shaped canal morphology in tooth groups where C-shaped canals were identified.

Age Group	MSM *n*/C-Shaped (%)	MTM *n*/C-Shaped (%)	MFP *n*/C-Shaped (%)	Total *n*/C-Shaped (%)	Chi-Square	Cramer’s V
18–24	46/3 (6.52%)	13/1 (7.69%)	64/0 (0.00%)	123/4 (3.25%)	χ^2^ = 3.999 *p* = 0.406	0.059
25–34	65/7 (10.77%)	71/3 (4.23%)	85/2 (2.35%)	221/12 (5.43%)		
35–44	135/7 (5.19%)	90/4 (4.44%)	109/1 (0.92%)	334/12 (3.59%)		
45–54	106/13 (12.26%)	103/4 (3.88%)	90/0 (0.00%)	299/17 (5.69%)		
55–64	64/4 (6.25%)	54/0 (0.00%)	40/0 (0.00%)	158/4 (2.53%)		
Total	416/34 (8.17%)	331/12 (3.63%)	388/3 (0.77%)	1135/49 (4.32%)		

MFP, mandibular first premolar; MSM, mandibular second molar; MTM, mandibular third molar; χ^2^, chi-square test; *p*, statistical significance with *p* value ≤ 0.05.

**Table 5:** Tooth-level symmetry of C-shaped canal morphology among identified C-shaped teeth.

	*n* Teeth	Symmetry *n* (%) Teeth	Asymmetry *n* (%) Teeth	Symmetry Scans	
				Identical *n* (%)	Non-Identical *n* (%)
MFP	3	2 (66.7)	1 (33.3)	0	1 (100)
MSM	34	18 (52.9)	16 (47.1)	3 (33.3)	6 (66.7)
MTM	12	4 (33.3)	8 (66.7)	0	2 (100)
Total	49	24 (49)	25 (51)	3 (25)	9 (75)

MFP, mandibular first premolar; MSM, mandibular second molar; MTM, mandibular third molar; *n*, number of teeth; symmetry indicates a C-shaped tooth on contralateral side; identical symmetry, matching canal configurations; non-identical, differing C-shaped canal morphologies on contralateral side.

**Table 6:** Patient tooth group bilateral occurrence of C-shaped canal morphology.

Tooth Group	C-Shaped Teeth *n*	Patients with ≥1 C-Shaped Tooth, *n*	Patients with Bilateral C-Shaped Teeth, *n*	Patients with Unilateral C-Shaped Teeth, *n*	Bilateral Occurrence Among C-Shaped Patients
MFP	3	2	1	1	1/2 (50.0%)
MSM	34	25	9	16	9/25 (36.0%)
MTM	12	10	2	8	2/10 (20.0%)
Total	49	37 tooth group patient observations	12 bilateral tooth group cases	25 unilateral tooth group observations	12/37 (32.4%)

MFP, mandibular first premolar; MSM, mandibular second molar; MTM, mandibular third molar. Note: Bilateral occurrence was calculated among patients with at least one C-shaped tooth in the corresponding tooth group. The total denominator of 37 represents tooth group patient observations, calculated as 2 MFP + 25 MSM + 10 MTM observations, and should not be interpreted as 37 patients because some patients have contributed C-shaped teeth in more than one tooth group. Therefore, the overall value of 12/37 (32.4%) represents descriptive bilateral occurrence among C-shaped tooth group observations, not true bilateral prevalence among all patients.

## Data Availability

The data will be made available upon sufficient request.

## Supplementary Materials

The following supporting information can be downloaded at: https://www.mdpi.com/article/10.3390/diagnostics16193127/s1, Table S1: STROBE checklist for reporting cross-sectional observational studies, Table S2: Distribution of evaluable and missing mandibular posterior teeth.

## Abbreviations

The following abbreviations are used in this manuscript:

3D	Three-Dimensional
CBCT	Cone Beam Computed Tomography
MFP	Mandibular First Premolar
MSP	Mandibular Second Premolar
MFM	Mandibular First Molar
MSM	Mandibular Second Molar
MTM	Mandibular Third Molar
