# Supplementary material for: C-Shaped Root Canal Morphology in Mandibular Posterior Teeth—A Retrospective CBCT Study Based on Two Classification Systems
Source: Diagnostics (Basel). 2026 Sep 26;16(19):3127. doi: 10.3390/diagnostics16193127 (PMC13664163; doi:10.3390/diagnostics16193127)
Supplement: Supplementary file 1 [file diagnostics-16-03127-s001.zip › diagnostics-4534281-supplementary.pdf]

Supplementary Table S1. STROBE checklist for reporting cross-sectional observational studies.

STROBE item	Recommendation	Location in manuscript	Reported/Action taken
**Title and abstract**	Indicate the study design and provide an informative summary of objectives, methods, results, and conclusions.	Title; Abstract	The study is described as a retrospective CBCT-based cross-sectional study.
**Background/rationale**	Explain the scientific background and rationale for the investigation.	Introduction	The Introduction explains the clinical importance of C-shaped canal morphology and the need for regional CBCT-based data.
**Objectives**	State specific objectives, including prespecified hypotheses where applicable.	End of Introduction	Objectives include prevalence, Fan classification, Ahmed and Dummer classification, bilateral occurrence, and gender comparison.
**Study design**	Present key elements of study design early in the manuscript.	Materials and Methods: Study design and setting	The study is identified as a retrospective cross-sectional observational study.
**Setting**	Describe setting, locations, and relevant dates, including periods of recruitment, exposure, follow-up, and data collection.	Materials and Methods: Study design and setting; Sample size and data collection	Study setting and CBCT scan period were standardized throughout the manuscript.
**Participants**	Give eligibility criteria, sources, and methods of selection of participants.	Materials and Methods: Inclusion and Exclusion Criteria	Inclusion/exclusion criteria were clarified.
**Variables**	Clearly define all outcomes, exposures, predictors, potential confounders, and effect modifiers.	Materials and Methods; Statistical Analysis	Variables included C-shaped canal presence, tooth type, age group, gender, side/quadrant, Fan classification, and Ahmed and Dummer classification.
**Data sources/measurement**	Describe data sources and measurement methods.	Materials and Methods: CBCT procedures and measurements	CBCT device parameters, software, assessment protocol, and observer calibration were described.
**Bias**	Describe efforts to address potential sources of bias.	Materials and Methods; Discussion/Limitations	Selection bias, retrospective design, missing teeth, and clustering limitations were acknowledged.
**Study size**	Explain how study size was determined.	Materials and Methods: Sample size and data collection	228 patients exceeded the estimated minimum of 139 independent patients.
**Quantitative variables**	Explain how quantitative variables were handled.	Statistical Analysis	Age was categorized into five groups: 18–24, 25–34, 35–44, 45–54, and 55–64 years.
**Statistical methods**	Describe all statistical methods, including those used to control confounding and subgroup analyses.	Statistical Analysis	Chi-square/Fisher's exact tests, Exact Monte Carlo tests, Holm–Bonferroni correction, Cramer's V effect sizes, and binomial regression were added.
**Missing data**	Explain how missing data were addressed.	Results; Discussion/Limitations	Missing/non-evaluable teeth were summarized in Supplementary Table 2, and discussed as a limitation in the manuscript.
**Participants/results flow**	Report numbers of individuals at each stage of study.	Results; Sample size and data collection	430 CBCT scans screened; 228 scans included; 1961 teeth evaluated.

**Descriptive data**	Give characteristics of study participants and information on exposures/confounders.	Results; Tables	Age-wise and patient-level summaries were added.
**Outcome data**	Report numbers of outcome events or summary measures.	Results; Tables 1–9 /Supplementary tables	Prevalence of C-shaped canals by tooth type, gender, side, age, and classification systems was reported.
**Limitations**	Discuss study limitations, direction and magnitude of potential bias.	Discussion/Limitations	Limitations include single-center retrospective design, small number of C-shaped cases, tooth-level clustering, lack of GEE/mixed-effects modeling, missing/extracted teeth, lack of sensitivity analyses, and paired right–left analysis limitations.
**Generalizability**	Discuss external validity of the study results.	Discussion/Limitations; Conclusions	Findings are limited to a Saudi subpopulation from one geographic region and may not be generalizable to all populations.
Funding	Give source of funding and role of funders.	Funding; Conflicts of Interest	Funding source and funder role were stated.

Supplementary Table S2. Distribution of evaluable and missing mandibular posterior teeth.

**Tooth type**	**Maximum teeth n**	**Evaluable teeth n**	**Missing teeth n (%)**
Mandibular first premolar	456	388	68 (14.9%)
Mandibular second premolar	456	403	53 (11.6%)
Mandibular first molar	456	423	33 (7.2%)
Mandibular second molar	456	416	40 (8.8%)
Mandibular third molar	456	331	125 (27.4%)
Total	2280	1961	319 (14.0%)

Note: The maximum possible number was calculated assuming two teeth per tooth type for each of the 228 patients/scans. Missing/non-evaluable teeth included absent, extracted, or otherwise non-assessable teeth based on the available CBCT records.
